# Effectiveness of virtual reality-based programs as vestibular rehabilitative therapy in peripheral vestibular dysfunction: a meta-analysis

**DOI:** 10.1007/s00405-023-07911-3

**Published:** 2023-03-22

**Authors:** Nagwa Mohamed Hazzaa, Ayat Farouk Manzour, Eman Yahia, Eman Mohamed Galal

**Affiliations:** 1 Audiology Unit, Otorhinolaryngology Department, Faculty of Medicine, Ain Shams University, Cairo, Egypt; 2 Community, Environmental and Occupational Medicine Department, Faculty of Medicine, Ain Shams University, Cairo, Egypt; 3 Audiology Unit, Toukh Central Hospital, Qalyubia, Egypt

**Keywords:** Virtual reality, Video games, Vestibular rehabilitation, Uncompensated peripheral vestibular dysfunction, Outcome measures, Meta analysis

## Abstract

**Purpose:**

To study the efficacy of virtual reality (VR) interventional programs as a vestibular rehabilitative method for patients with uncompensated peripheral vestibular disorders.

**Methods:**

The databases PubMed, Google scholar, Embase and Cochrane Library were used (up to July 2021). Studies selected in this study were controlled trials in which virtual reality was used as vestibular rehabilitative therapy in comparison to any other vestibular rehabilitative methods or medical or dietary recommendations. Comparison was made in at least one of these outcomes measures; Subjective measures such as Dizziness Handicap Inventory, Vertigo Symptom Scale—Short Form questionnaire, Activities-specific Balance Confidence questionnaire, Dizziness Analogue Scale or Visual Analogue Scale, besides objective measures as posturography. Six articles were included in the meta-analysis; tested for heterogeneity of the estimates using chi-squared and I2 tests, outcomes were expressed as mean difference and 95% CI. Estimates from included studies were pooled using the random-effect model.

**Results:**

virtual reality as a vestibular rehabilitative intervention was able to improve scores of Dizziness Handicap Inventory, Vertigo Symptom Scale—Short Form questionnaire, Visual Analogue sale and posturography as outcome measures of vestibular rehabilitation.

**Conclusion:**

virtual reality has a potential clinical benefit for vestibular rehabilitation in peripheral vestibular dysfunction compared with conventional vestibular rehabilitation methods. However, further research is needed to document the exact parameters of an optimal protocol for virtual reality rehabilitation, the period needed for effective rehabilitation and its side effects.

## Introduction

Vestibular rehabilitation (VR) therapy is considered a safe and effective treatment for uncompensated peripheral vestibular dysfunction [1]. It can also improve static and dynamic balance and gait, as well as reduce comorbid symptoms of dizziness such as depression and anxiety, resulting in an increase in patients' self-confidence and quality of life [2]. Vestibular rehabilitation integrates proprioceptive, visual, and residual vestibular function to improve balance, including gaze, gait and postural stability, and physical mobility [3]. However, many factors may negatively affect the outcome of vestibular rehabilitation, including incorrect exercises performance and the necessity of active efforts and motivation of the patient [4].

VR-based vestibular rehabilitative therapy is one of the most innovative and promising recent developments in rehabilitation technology in which the users interact with displayed images, move and manipulate virtual objects and perform other actions in a way that attempts to "immerse” them within the simulated environment engendering a feeling of presence in the virtual world [5]. VR systems can be equipped with real-time simulation, interactive functions, and game features enabling adaptation, habituation, and substitution exercises to fulfill vestibular rehabilitation objectives [6].

With complete control over the stimuli presented to the subject, VR offers a standardized and reproducible setting for vestibular rehabilitation that has several benefits [7]. Additionally, the facts that VR is a pleasurable training tool motivate patients to continue their rehabilitation [8]. Compared to traditional rehabilitative therapy approaches, it provides more feedback, stimulating and enriching development [9]. Additionally, it improves gait capability, lower extremity function, and consequently balance [10].

Meanwhile, there are some limitations of virtual reality such as the latency of the system; all the steps from the capture of information (motion, center of pressure, etc.) to the multisensory feedback (i.e., visual) take time, this delay can be perceived by the patient, consequently he may modify his reactions, so there is a different perception of distances and underestimation in VR compared to the real situation [8]. In addition, Cyber-sickness experienced, especially with the more immersive virtual environments [11]. Nausea, vomiting, headache, somnolence, loss of balance, and altered eye–hand coordination are all common symptoms of cyber-sickness [12]. Sometimes the inconsistency between the information received by the body and the eyes in virtual reality leads to dizziness, headache and sickness [13].

However, many studies were done exploring the efficacy of VR as a rehabilitative tool, but no standardized guidelines have been settled in the literature and there is also a high diversity of settings and protocols involving virtual reality for vestibular rehabilitation. Accordingly, this study aimed to search, critically appraise, and synthesize the best available evidence on whether virtual reality interventions are effective in the rehabilitation of peripheral vestibular dysfunction or not. This research systematically reviewed the previous studies to summarize the published protocols documenting the use of virtual reality settings for peripheral vestibular disorders rehabilitation.

## Methods

This systematic review was performed following the (Preferred Reporting Items for Systematic Reviews and Meta-Analyses) PRISMA 2020 guidelines [14].

### The criteria for considering studies

Studies included in this study are the controlled trials in which VR was used as vestibular rehabilitative therapy in comparison to any other vestibular rehabilitative (conventional) modalities, medical treatment or dietary recommendations.

Research question: Is virtual reality-based vestibular rehabilitation therapy effective in the rehabilitation of uncompensated peripheral vestibular dysfunction? This research question was established following recommendations from the PICO format (Participants, Intervention, Comparison and Outcome measures) [15].

- Type of participants: Included male and female patients, over 18 years of age, clinically diagnosed with unilateral or bilateral peripheral vestibular dysfunction with no specifically diagnosed neurological disorder.
- Type of interventions: Balance, gaze and gait training using any virtual reality system, with no restrictions on the technique used in rehabilitation or duration of the rehabilitation.
- Comparison: virtual reality methods were compared to any other vestibular rehabilitative methods as well as medical treatment or dietary recommendations.
- Types of outcome measures: In the studies, at least one of the following outcome measures required to be used to compare the experimental and control groups:
- Subjective measures such as Dizziness Handicap Inventory (DHI), Vertigo Symptom Scale—Short Form questionnaire (VSS-SF), Activities-specific Balance Confidence questionnaire (ABC), Dizziness Analogue Scale or Visual Analogue Scale (VAS) (SAT) Score of patient satisfaction.
- Objective measures such as posturography.

Exclusion criteria included studies without a comparison group (as case studies, observational studies etc.), studies with participants younger than 18 years, patients with central or mixed causes vestibular dysfunctions**, s**tudies with target outcome other than those mentioned previously in inclusion criteria, studies comparing two types of virtual reality, as well as studies involving healthy subjects and studies not in the English language.

### Research method

Review process included procedures, considerations, and decisions that lead to a consolidated list of articles to be reviewed in-depth. This article review process consisted of the following steps;

#### Definition of the review scope, keywords, and research question

Although the research questions have been identified, defining the scope and keywords was quite challenging because, in fact, VR research is extensive and the number of publications in this area is abundant.

The search was done by extracting key information from documents indexed in four scientific digital libraries; PubMed, Google Scholar, Cochrane and Embase.

#### Initial paper search.

The search was conducted using MeSH (Medical Subject Headings) by changing between the following keywords: (virtual reality—video games—vestibular rehabilitation—peripheral vestibular dysfunction) as shown in Table 1, and the terms were used as keywords in the title and abstract in all databases, with one or more concept of these keywords, the simple or advanced search when possible was conducted using the Boolean operators ‘‘AND’’ and ‘‘OR”. These databases were searched until July 2021.

**Table 1:** Shows the different keywords used in the electronic databases survey

Search concept 1	Search concept 2	Search concept 3	Search concept 4
Efficacy	Virtual reality	Vestibular rehabilitative therapy	Peripheral vestibular dysfunction
Effect	Virtual video-games	Vestibular rehabilitationon	Peripheral vestibular lesion
Role of	Computer games		Bilateral or unilateral peripheral vestibular dysfunction

#### Removing duplicate documents

Once the papers were identified; the duplicated studies were removed using the EndNote program [computer program], Version X9, Clarivate Analytics, 2018.

#### Manual filtering by reading the titles and abstracts

Articles were selected by two independent reviewers who analyzed the title and the abstract. If there were disagreements, the remaining two authors would also judge the abstracts.

#### Manual filtering by reading the contents and excluding irrelevant entries

At this stage, the full text was analyzed, the results were compared, and discrepancies between articles were discussed.

### Data collection methods

This was created manually by the authors by summarizing the information for qualitative and quantitative synthesis from the included studies by using Excel files. The study characteristics (the author's name, year of publication, journal, site in which the study was done, comparison groups, type of intervention, measurements of efficacy, and outcome measures) were determined. Any disagreements on data extraction or quality assessment were resolved by consensus between the authors.

### Data management and analysis methods

Data was revised for completeness and consistency. The program used for building the meta-analysis model was Review Manager (RevMan) [Computer program], Version 5.4, The Cochrane Collaboration, 2020. The used models were tested by random effect. The graphical presentation was extracted from Review Manager 5.4.

Studies included in the meta-analysis were tested for heterogeneity of the estimates using the following tests: the chi-squared (*χ*^2^, or Chi^2^), the *I*^2^ test and the *p* value. A forest plot was used to display effect estimates and confidence intervals for both individual studies and meta-analyses .

## Results

The search in the databases; PubMed, Google Scholar, Embase, and Cochrane Library, (up to July 2021) yielded 501 articles.

**Fig. 1:**
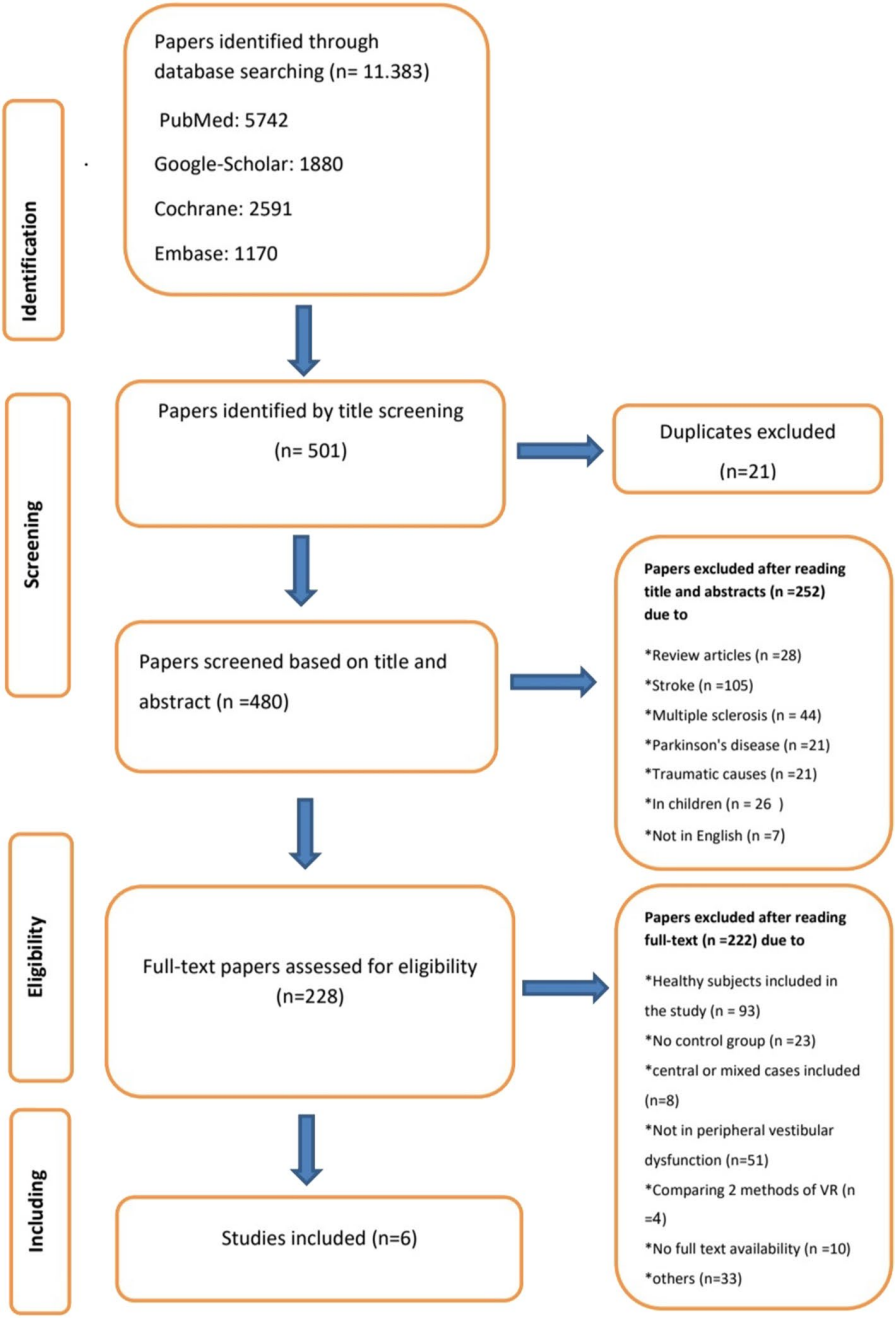
The flowchart shows the research and screening process for article inclusion

After removing duplicates, 480 articles remained. Of these, 252 were excluded based on the title and abstract due to various causes as shown in Fig. 1; studies focused on other diseases such as stroke, multiple sclerosis, and Parkinson’s disease. Other studies focused on traumatic causes of imbalance. Studies that included children, studies not in the English language and review articles were also excluded. After reading the full text of 228 articles; 222 were excluded, due to the presence of normal subjects in the studies, the presence of central or mixed vestibular cases, Alzheimer, acute vestibular neuritis or undetected causes of fall or imbalance. Also uncontrolled studies, studies compared two types of virtual reality, studies without proposed outcomes and studies in which virtual reality was used as a diagnostic tool were also excluded. The full text of some studies was not available so they were excluded.

Finally, six articles were included in our study as shown in the flowchart in Fig. 1.

## Summary of studies

Two hundred fifty-eight patients participated in the included six studies. Patients were diagnosed with peripheral vestibular lesions whether unilateral or bilateral.

A summary of these studies can be found in the following Tables 2, 3, 4.

**Table 2:** This table shows a summary of the six included studies regards the name of the 1st author, year of publication, Journal of publication and the site of the study, diagnosis, number of patients, mean age and male-to-female ratio in experimental and control groups

No.	Author	Year	Diagnosis Site of study Journal	Number of participants			Mean age (SD) in years		Male: female ratio	
				Total number subjects	Experimental group	Control group	Experimental group	Control group	Experimental group	Control group
1	Garcia et al. [16]	2013	Ménière’s disease (unilateral or bilateral) São Paulo-Brazil Brazilian Journal of Otorhinolaryngology	44	23	21	47.65	47.90	9:14	7:14
2	Meldrum et al. [17]	2015	Unilateral peripheral vestibular lesion Dublin-Ireland Archives of Physical Medicine and Rehabilitation Journal	71	36	35	57.83 ± 13.6	50.47 ± 15.53	14:21	13:23
3	Micarelli et al. [18]	2017	Unilateral peripheral vestibular lesion Rome-Italy International Journal of Rehabilitation Research	47	23	24	49.72 ± 10.34	50.47 ± 15.53	14:9	13:11
4	Phillips et al. [19]	2018	Unilateral peripheral vestibular lesion East Anglia-UK The Journal of Laryngology & Otology	26	14	12	48 ± 15	47 ± 16	6:15	8:11
5	Rosiak et al. [20]	2018	Unilateral peripheral vestibular lesion Lodz, Poland Journal of Vestibular Research	50	25	25	46.48 ± 10.6	45.20 ± 11.07	11:4	12:13
6	Stankiewicz et al. [21]	2020	Unilateral peripheral vestibular lesion Lodz, Poland International Medical Journal of Experimental and Clinical Research	20	10	10	49.7	48.2	6:4	5:5

**Table 3:** Continued summary of included studies regards intervention in experimental and control groups

Author, year	Intervention in experimental group	Intervention in control group
1 Garcia et al. (2013)	Virtual reality stimuli in a Balance Rehabilitation Unit (BRUTM) {45 min twice a week for 6 weeks (total of 12 sessions)}** + **dietary recommendations and prescribed 48 mg/day of betahistine	Dietary recommendations and prescribed 48 mg/day of betahistine
2 Meldrum et al. (2015)	Balance activities using the Wii Fit Plus {15 min/day for 5 days a week for 6 weeks} total of 30 sessions + gaze stabilization exercises + walking program	Balance activities using a foam mat {15 min/day for 5 days a week for 6 weeks} + gaze stabilization exercises + walking program
3 Micarelli et al. (2017)	The Track Speed Racing 3-D game with a head-mounted display + Traditional vestibular rehabilitation activities (a combination of adaptation, substitution, habituation, and balance exercises) {30 to 45-min/twice per week for 4 weeks} total of 8 sessions Between sessions, patients did a twice-daily home exercise program for a total of 30–40 min/day	The traditional vestibular rehabilitation activities (a combination of adaptation, substitution, habituation, and balance exercises) {30 to 45-min/twice per week for 4 weeks} Between sessions, patients did a twice-daily home exercise program for a total of 30–40 min/day
4 Phillips et al. (2018)	Wii Fit computer games with a balance platform (9 different balance games were used) {two 30-min sessions per day for 16 weeks}	Traditional vestibular rehabilitation activities (standard customized vestibular rehabilitation protocol) {two 30-min sessions per day for 16 weeks}
5 Rosiak et al. (2018)	A Virtual Reality-based exercise program using a hybrid VR unit {10 training sessions over 2 weeks, session last for 30 min} + Cawthrone-Cooksey exercises at home three times daily	Static posturography with visual feedback training {10 training sessions over 2 weeks, session last for 25 min} + Cawthrone-Cooksey exercises at home three times daily
6 Stankiewicz et al. (2020)	Vestibular rehabilitation using virtual reality ‘VR Roller Coaster’ application {2 sessions of 5 min with 5-min intervals for 5 consecutive days} + conventional Cawthorne-Cooksey exercises {5 sessions for 5 consecutive days}	Conventional Cawthorne-Cooksey exercise {5 sessions for 5 consecutive days}

**Table 4:** Continued summary of included studies regards time of assessment and outcome measures

Author	Time of assessment	Outcome measures
1 Garcia et al. (2013)	Before intervention After a 6-weeks period of treatment	1 The Dizziness Handicap Inventory (DHI) [22] 2 Dizziness analog scale 3 Posturography—center of pressure (COP) for analysis “BRUTM” [23]
2 Meldrum et al. (2015)	Before intervention After an 8 weeks of treatment After a 6 months of treatment	1 Gait speed 2 Dynamic visual acuity (DVA) 3 Activities Balance Confidence Scale [24] 4 The Vestibular Rehabilitation Benefits Questionnaire 5 Hospital Anxiety and Depression Scale
3 Micarelli et al. (2017)	Before intervention After a 4-weeks period of intervention	1 The Italian Dizziness Handicap Inventory 2 Static Posturography Testing 3 Video Head Impulse Testing 4 The Activities Balance Confidence Scale [24] 5 Simulator Sickness Questionnaire (SSQ)
4 Phillips et al. (2018)	Before the intervention Further assessments were made at 4, 8, 12 and 16 weeks (VAS was only completed at weeks 4 and 16)	1 The Dizziness Handicap Inventory (DHI) [25] 2 The Short Form 36 quality of life questionnaire (SF36) 3 Visual analogue scale (VAS) to assess the enjoyment experienced whilst performing the exercises
5 Rosiak et al. (2018)	Before intervention One month after intervention	1 Posturographic assessment on a static platform (the COP used for analysis) 2 Vertigo Symptom Scale—Short Form (VSS-SF) [26]
6 Stankiewicz et al. (2020)	Before the intervention 4 weeks after the end of the study VAS scale done as well as at each therapeutic visit	1 Vertigo Symptom Scale—Short Form (VSS-SF) [26] 2 Visual Analog Scale (VAS) evaluating patient satisfaction (SAT) 3 Dizziness analog scale

### Pooling of estimates

Outcomes were expressed as mean difference (MD) and 95% confidence interval (CI) Due to the presence of some heterogeneity between the studies in some scales as determined by the chi-squared test and* I*^2^ test, the data was analyzed using the random effect model as it is the more conservative approach even if there is no heterogeneity. In cases of high unaccepted heterogeneity between the included studies, the results of the meta-analysis were insignificant so qualitative analysis had to be done.

#### Posturography (COP) on firm surface with closed eye

See Fig. 2.

**Fig. 2:**
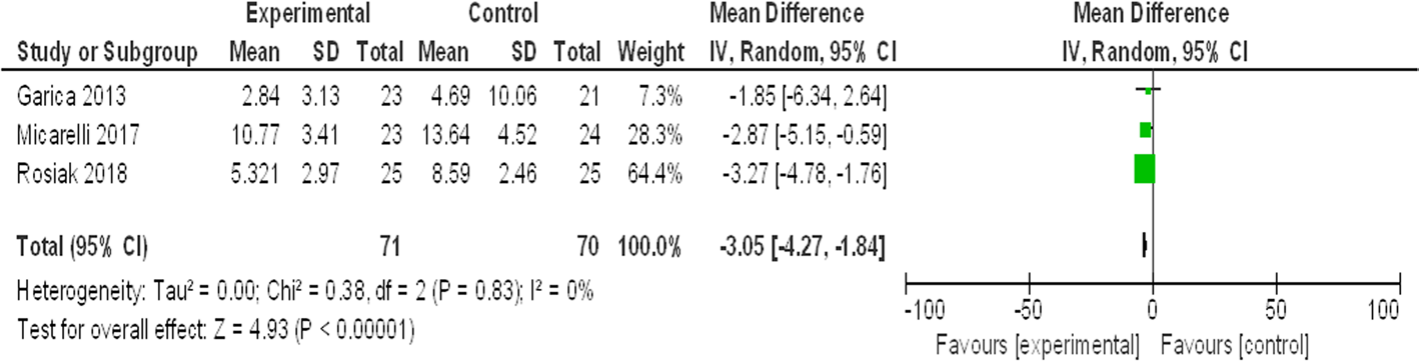
Forest plot for meta-analysis of posturography (COP on a firm surface with a closed eye)

#### Vertigo Symptom Scale Short Form (VSS-SF)

See Fig. 3.

**Fig. 3:**
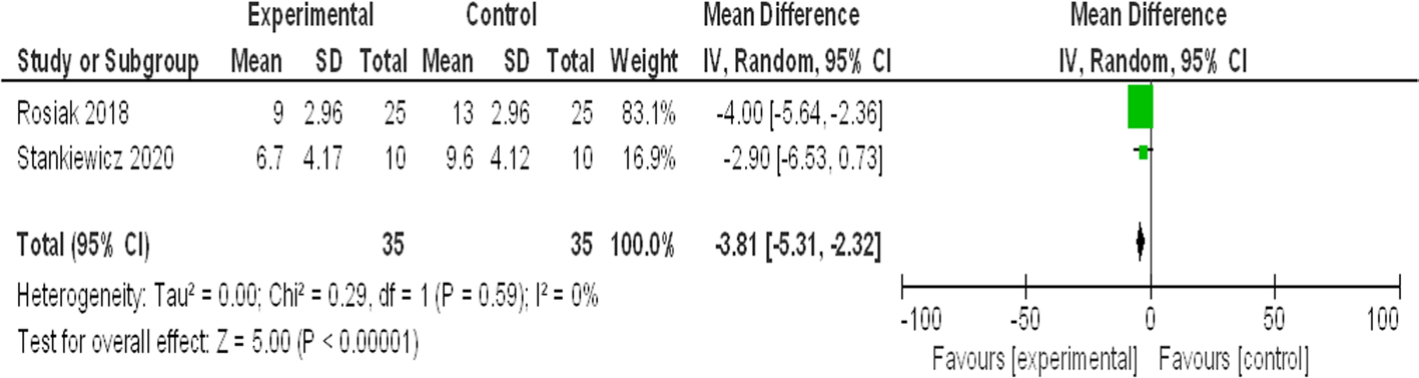
Forest plot for meta-analysis of Vertigo Symptom Scale Short Form (VSS-SF)

#### Visual analogue scale (VAS) (SAT) score of patient satisfaction

See Fig. 4.

**Fig. 4:**
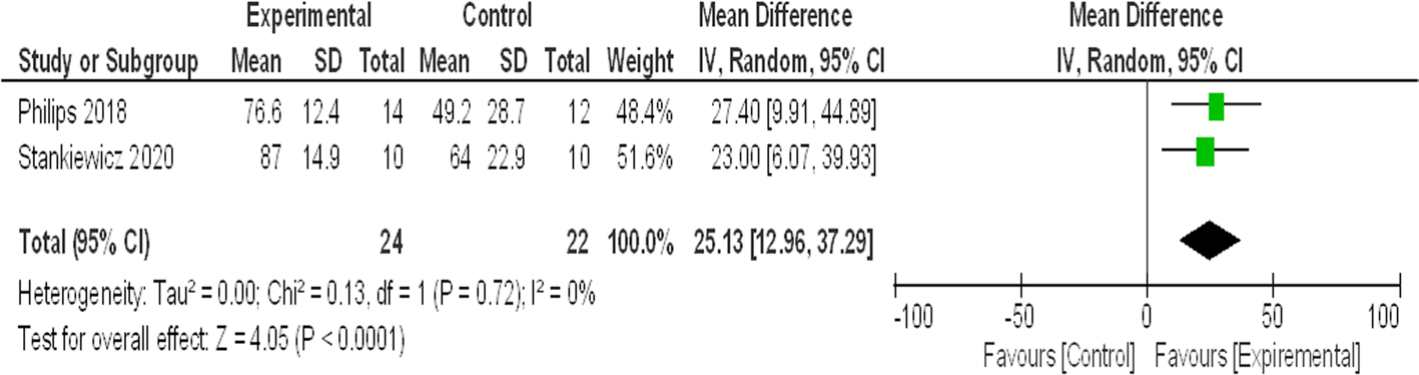
Forest plot for meta-analysis of VAS (SAT) Score

#### Dizziness Handicap Inventory (DHI) total score

See Fig. 5.

**Fig. 5:**
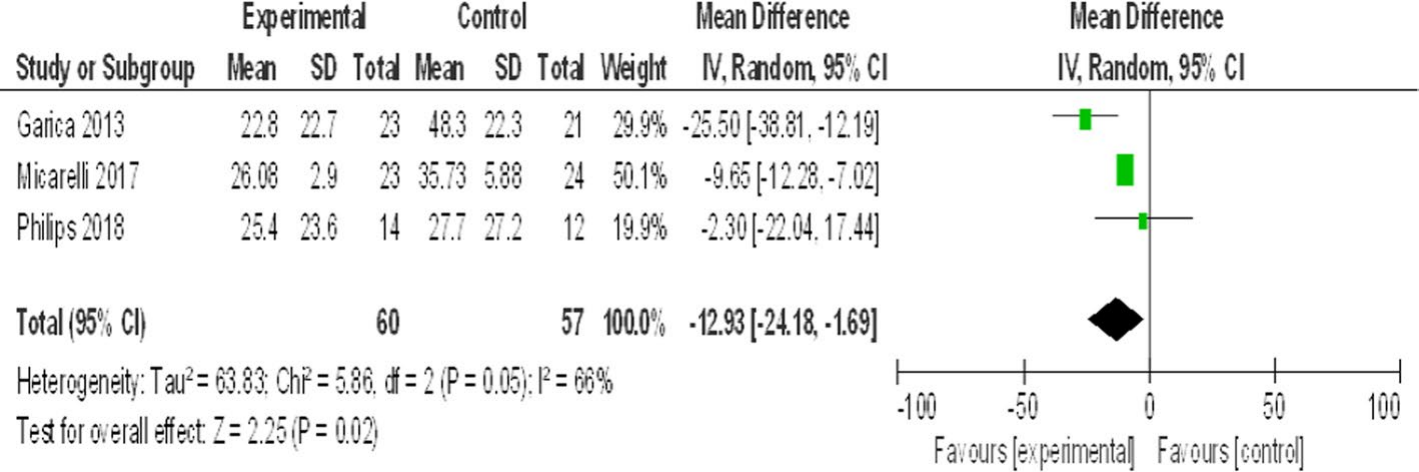
Forest plot for meta-analysis of Dizziness Handicap Inventory (DHI) total score

#### Activities-specific balance confidence (ABC) scale

See Fig. 6.

**Fig. 6:**
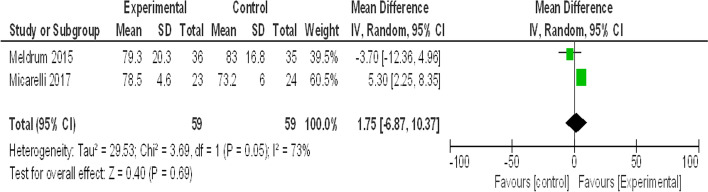
Forest plot for meta-analysis of Activities specific Balance Confidence (ABC)

#### Dizziness analogue scale (DAS)

**Fig. 7:**
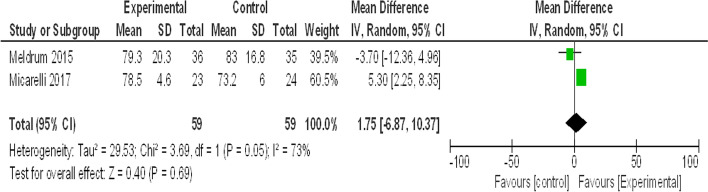
Forest plot for meta-analysis of Dizziness analogue scale

See Fig. 7 and Table 5.

**Table 5:** Summary of significance of outcome measures meta-analysis

Outcome measures	*P* value	Significance
Posturography	˂ 0.00001	Highly significant
VSS-SF	˂ 0.00001	Highly significant
VAS (SAT)	˂ 0.00001	Highly significant
DHI total score	= 0.02	Significant
ABC scale	= 0.69	Non-significant
Dizziness analogue scale	= 0.10	Non-significant

## Discussion

The aim of this study is to study the efficacy of virtual reality-based vestibular rehabilitation in patients with peripheral vestibular disorders. There are previously published systematic reviews that evaluated this issue; Bergeron et al. [27], demonstrated the promising potential of VR in vestibular rehabilitation, despite the significant differences in terms of protocols used and outcome evaluation among their selected studies. They mentioned that none of the selected seven studies had low methodological quality; as four studies only had a control group. In addition, few studies used validated questionnaires, so direct and standardized comparison between studies was not applicable. Meanwhile, they concluded that time spent in VR-based training contributed more in its efficiency than the number of sessions. So, longer sessions in a shorter period of time could be effective and convenient.

In 2019, Kinne et al. [28] conducted a systematic review to evaluate the efficacy of home-based virtual reality systems on vestibular rehabilitation outcomes, concluding that these interventions effectively achieved the primary objectives of vestibular rehabilitation; but the use of these interventions was equally as effective as the use of traditional vestibular rehabilitation program. In addition, they reported that it may be most beneficial to combine home-based virtual reality with traditional vestibular rehabilitation methods.

Heffernan et al. [29] included five randomized controlled trials in their review. Meta-analysis was done on 4 of them using the DHI total score only, concluding that virtual reality vestibular rehabilitation improves DHI scores significantly more than conventional vestibular rehabilitation alone 0–3 months’ post-intervention.

Regarding the current meta-analysis, the authors tried to improve some of the methodological aspects of the previous systematic reviews. The six studies included in this study were controlled studies, all of them were randomized except Rosiak et al. [20] which was a non-randomized controlled trial. This helps to increase the level of evidence of the included studies. Also objective as well as subjective outcome measures were used. The use of the objective measure revealed more strength to the current study; as although subjective outcomes are more important in establishing clinical benefit, evidence of similar improvements in objective outcomes can enhance the internal validity of the studies.

This study attempts to synthesize the results of the included studies into one pooled estimate providing a meta-analysis. They included two hundred fifty-eight (258) patients diagnosed with peripheral vestibular lesions (unilateral in 5 studies and bilateral in 1 study).

The meta-analysis performed in this study on posturography (COP on a firm surface with a closed eye) (Fig. 2) was reported by 3 of the included studies. The test of heterogeneity is 0%, which shows no heterogeneity and the P value of the overall effect estimate is highly significant (*P* ˂ 0.00001). So, virtual reality is better than the other methods in improving postural control and increasing stability limits. This was explained as VR improves the vestibular source of spatial information and subsequent central reweighting and related postural control [18].

Regarding the subjective outcomes; VSS-SF, VAS- SAT and DHI total score, their meta-analyses revealed that virtual reality is superior with high significance (*P *˂ 0.00001) to the traditional vestibular rehabilitation activities as shown in Figs. 3, 4 and 5. VSS-SF reduced scores were as a result of decreased overall sensation of dizziness (physical and emotional states), intensity decreased more with the virtual reality group. Furthermore, the Visual Analog Scale (VAS)—SAT revealed that patients in virtual reality groups were more satisfied with the VR-rehabilitative method. Virtual reality is characterized as being an enjoyable and easily accessible form of therapy. The test of heterogeneity is 0% (showing no heterogeneity), and the *P* value of the overall effect estimate is highly significant (*P *˂ 0.0001). Additionally, Stankiewicz et al. [21] added that the beneficial therapeutic effects are experienced earlier with VR therapy than the conventional methods.

On the other hand, the Activities-specific balance confidence (ABC) scale that was reported by two of the included studies, Meldrum et al. and Micarelli et al.. In Meldrum et al. [17], there was no significant difference between the virtual reality group and the traditional vestibular rehabilitative group for the ABC scale. Although their patients reported VR was more enjoyable, less fatigable and less difficult in balance exercises, taking in consideration that Meldrum et al. used a home-based VR program. While in Micarelli et al. [18], there was a significant post-treatment effect change in the ABC scale which is in agreement with the systematic review Xie et al. [30].

While there was a significant post-treatment change in the ABC scale in Micarelli et al. [18], this is consistent with the systematic review by Xie et al. [30]. Accordingly, there was substantial high heterogeneity (73%) between the results of the ABC scale between these two included studies and the results of pooling of their estimates were not significant (*P* = 0.69) (Fig. 6).

The meta-analysis performed on the Dizziness Analogue Scale (DAS) that was used by two of the included studies, Garcia et al. and Stankiewicz et al., revealed that the virtual reality group showed more significant score reductions in the Dizziness analogue scale and, therefore, improvements from dizzy spells when compared against controls. But there was moderate heterogeneity (58%), and the results of the pooling of estimates were not significant (*P* = 0.10) (Fig. 7).

Side effects of virtual reality interventions were studied by Micarelli et al. using the validated simulator sickness questionnaire (SSQ). They examined how the patients were habituated to the intervention at the end of each week of rehabilitation. Symptoms decreased over time; there was a significant decrease in nausea, oculomotor stress, and disorientation scores from the first to the fourth week of the VR rehabilitation program. They suggested that patients were safely habituating to the virtual reality stimuli. Also, Pavlou et al. [31] revealed nearly the same results. While Meldrum et al. [17] found low back pain, neck pain, and severe nausea (in 2/35 patients).

No study reported major side effects following the use of virtual reality and no significant incident or fall was reported.

### Criticism for the included studies

The games used in the virtual reality programs were not fully described as in Meldrum et al. [17] and Phillips et al. [19]. Also, most of the studies did not monitor or report the side effects of the rehabilitation programs, and some studies did not document clearly the time, and a number of sessions spent in rehabilitation or the time between sessions.

## Conclusion

This systematic review and meta-analysis show that virtual reality as a vestibular rehabilitation measure has potential clinical benefit as it improves DHI, VSS-SF, VAS-SAT and posturography parameters in peripheral vestibular disorders, besides providing more satisfaction and pleasure than the conventional rehabilitative methods.

## Recommendations

Further research is needed to document the exact parameters of an optimal protocol, the period needed for effective rehabilitation, and the number of sessions, as well as report the side effects of virtual reality in patients with peripheral vestibular dysfunction.

## Declarations

### Submission declaration and verification

This work has not been published previously.

### Availability of data and material

The datasets generated and/or analyzed during the current study are available from the corresponding author upon reasonable request.
